# Homoserine Lactones Influence the Reaction of Plants to Rhizobia

**DOI:** 10.3390/ijms140817122

**Published:** 2013-08-20

**Authors:** Azhar A. Zarkani, Elke Stein, Christian R. Röhrich, Marek Schikora, Elena Evguenieva-Hackenberg, Thomas Degenkolb, Andreas Vilcinskas, Gabriele Klug, Karl-Heinz Kogel, Adam Schikora

**Affiliations:** 1 Institute of Phytopathology and Applied Zoology, Centre for BioSystems, Land Use and Nutrition, Justus Liebig University Giessen, Heinrich-Buff-Ring 26-32, D-35392 Giessen, Germany; E-Mails: azhar.a.zarkani@agrar.uni-giessen.de (A.A.Z.); elke.stein@agrar.uni-giessen.de (E.S.); thomas.degenkolb@ernaehrung.uni-giessen.de (T.D.); andreas.vilcinskas@agrar.uni-giessen.de (A.V.); karl-heinz.kogel@agrar.uni-giessen.de (K.-H.K.); 2 Fraunhofer Institute for Molecular Biology and Applied Ecology (IME), Bioresources Project Group, Winchesterstrasse 2, D-35394 Giessen, Germany; E-Mail: christian.r.roehrich@fhg.uni-giessen.de; 3 Department Sensor Data and Information Fusion, Fraunhofer FKIE, 53343 Wachtberg, Germany; E-Mail: marek.schikora@fkie.fraunhofer.de; 4 Institute of Microbiology and Molecular Biology, Centre for BioSystems, Land Use and Nutrition, Justus Liebig University Giessen, Heinrich-Buff-Ring 26-32, D-35392 Giessen, Germany; E-Mails: elena.evguenieva-hackenberg@mikro.bio.uni-giessen.de (E.E.-H.); gabriele.klug@mikro.bio.uni-giessen.de (G.K.)

**Keywords:** quorum sensing, induced resistance, plant-bacteria interaction, homoserine lactones

## Abstract

Bacterial quorum sensing molecules not only grant the communication within bacterial communities, but also influence eukaryotic hosts. *N-*acyl-homoserine lactones (AHLs) produced by pathogenic or beneficial bacteria were shown to induce diverse reactions in animals and plants. In plants, the reaction to AHLs depends on the length of the lipid side chain. Here we investigated the impact of two bacteria on *Arabidopsis thaliana*, which usually enter a close symbiosis with plants from the Fabaceae (legumes) family and produce a long-chain AHL (*Sinorhizobium meliloti*) or a short-chain AHL (*Rhizobium etli*). We demonstrate that, similarly to the reaction to pure AHL molecules, the impact, which the inoculation with rhizosphere bacteria has on plants, depends on the type of the produced AHL. The inoculation with oxo-C14-HSL-producing *S. meliloti* strains enhanced plant resistance towards pathogenic bacteria*,* whereas the inoculation with an AttM lactonase-expressing *S. meliloti* strain did not. Inoculation with the oxo-C8-HSL-producing *R. etli* had no impact on the resistance, which is in agreement with our previous hypothesis. In addition, plants seem to influence the availability of AHLs in the rhizosphere. Taken together, this report provides new insights in the role of *N-*acyl-homoserine lactones in the inter-kingdom communication at the root surface.

## 1. Introduction

Many Gram-negative bacteria use *N-*acyl-homoserine lactones (AHLs) for their intra-population communication. Such communication between bacterial individuals, termed quorum sensing (QS), was discovered more than 40 years ago [1,2]. Today, we understand very well the mechanisms of QS within the bacterial populations. Notably, QS molecules used by beneficial or pathogenic bacteria have also impact on the eukaryotic host. The first indication that QS-molecules of rhizosphere bacteria influence plant defense responses came from a study on the interaction between *Serratia liquefaciens* MG1 and tomato (*Solanum lycopersicum*) [3]. *Serratia liquefaciens* MG1 produces C4- and C6-homoserine lactones when colonizing the root surface [4]. Colonization of the root surface with *S. liquefaciens* induced systemic resistance against the leaf-pathogenic fungus *Alternaria alternata* in tomato*,* whereas the AHL-negative *S. liquefaciens* mutant MG44 was not able to induce such resistance [3]. In a similar manner, colonization with the AHL-producing *Serratia plymuthica* wild-type strain HRO-C48 protected cucumber plants (*Cucumis sativus*) from the damping-off disease caused by the oomycete *Pythium aphanidermatum,* as well as tomato and bean (*Phaseolus vulgaris*) from infection with *Botrytis cinerea*, the causing agent of gray mold [5]. Similar to the study with *S. liquefaciens* MG44 and tomato, the authors demonstrated that the *splI*^−^ mutant of *S. plymuthica*, impaired in the production of AHLs, could not provide protection against *P. aphanidermatum* and *B. cinerea*. These results provide evidence that AHLs play an important role in the induction of plant defences. However, contradictory results were reported for the interaction between *Arabidopsis thaliana* and *S. liquefaciens* MG1 and its AHL-negative mutant MG44 [6]. Because the resistance against the pathogenic bacterium *Pseudomonas syringae* on *A. thaliana* leaves was not differently induced by the *S. liquefaciens* wild type and its AHL-negative mutant, the authors suggested an AHL-independent resistance increasing effect against *P. syringae* caused by root colonization with *S. liquefaciens* [6]. Thus, bacteria-plant interaction experiments with living microbial cells have to be interpreted very carefully, since different bacteria can induce different plant responses independently of the QS auto-inducer system. A helpful mean to circumvent this disturbing overlap effect was the use of pure AHL-compounds.

AHLs vary in the length of the lipid side chain and the substitution on the C_3_-atom (O- or OH-group). The length of the lipid side chain is important for the reaction of plants. C4-HSL, C6-HSL, oxo-C6-HSL and oxo-C8-HSL promoted growth of *Arabidopsis* [6–8]. Oxo-C10-HSL induced the formation of adventitious roots in mung beans [9]. On the other hand, oxo-C14-HSL and to a lesser extend OH-C14-HSL induced resistance in *Arabidopsis* and barley plants towards biotrophic and hemibiotrophic pathogens [10]. Likewise, oxo-C12-HSL has a resistance-inducing potential, though weaker than C14-HSL derivatives [8]. Comparison of five different AHLs differing in the length of their lipid side chain, which ranged from 6 to 14 carbons, on plant growth revealed clear differences.

We here report the effect of long-chain oxo-C14-HSL-producing *Sinorhizobium meliloti* on plant resistance. We show that the effect is dependent on the presence and type of the AHL, since only the inoculation with *S. meliloti* strains producing oxo-C14-HSL had a positive effect on *Arabidopsis* resistance towards pathogenic bacteria. In contrast, inoculation with an AHL-negative *S. meliloti* strain or an oxo-C8-HSL-producing *Rhizobium etli* was not able to enhance resistance. Remarkably, the impact of AHLs on plant resistance is independent of the native host-symbiont system, as shown by the influence on *Arabidopsis*, which is not forming nodules and not a symbiotic host for both *S. meliloti* and *R. etli*. In addition, our results suggest that plants influence the amount of QS molecules in the rhizosphere and therefore might interfere with the bacterial intercellular communication.

## 2. Results

### 2.1. *S. meliloti* Rm2011 Produces *N*-3-Oxo-Tetradecanoyl-l-Homoserine Lactone (oxo-C14-HSL)

**Figure 1:**
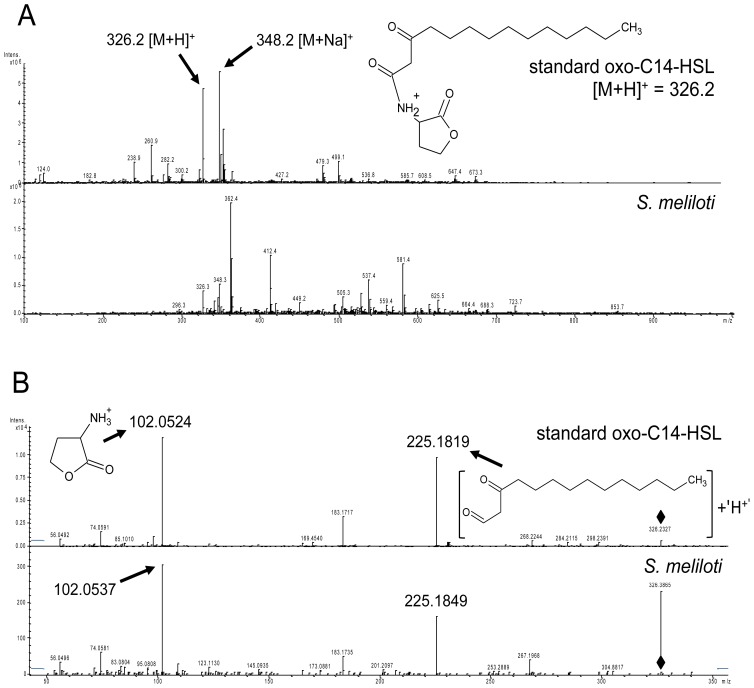
Verification of *N-*acyl-homoserine lactone (AHL) production in *S. meliloti* Rm2011. HPLC samples were screened in the positive ion mode from *m/z* 50 to 400. (**A**) The MS of standard oxo-C14-HSL standard (*m*/*z* = 326.2 [M + H]^+^) shows a pattern similar to the AHL extracted from *S. meliloti* Rm2011 culture; (**B**) Further analysis of *m*/*z* 326.2 by MS/MS confirmed the identity of AHL extracted from *S. meliloti* Rm2011 culture with the standard oxo-C14-HSL. The diagnostic fragment ions *m*/*z* 102.0524 and 102.0537 are indicative for the presence of the lactone ring. The highlighted *m*/*z* values represent diagnostic fragment ions. Diamonds mark the pseudomolecular ions used as precursors for MS/MS analysis.

In our previous report we showed that long-chain AHLs (e.g., oxo-C14-HSL) induce resistance in *A. thaliana* towards hemibiotrophic and biotrophic pathogens [10]. *S. meliloti* produces different long-chain AHLs [11] and was therefore a good candidate to study the interaction between *A. thaliana* and long-chain AHLs produced by rhizosphere bacteria. To commence this study*,* we determined which AHLs are synthesized in our conditions by *S. meliloti* Rm2011, a natural mutant with insertion in one of the AHL receptor genes, *expR* [12]. In order to define the type of AHLs, we used an LC-MS/MS approach. Bacteria were grown in 80 ml of TY medium [13] and the cell-free culture supernatant was used for the analysis. As standards, we used commercially available C6-, oxo-C8-, oxo-C10-, oxo-C12-, and oxo-C14-HSL. We detected oxo-C14-HSL (Figure 1), confirming the previous results with other *S. meliloti* strains including the closely related strain Rm1021, which is also an *expR* mutant [12]. The identity of oxo-C14-HSL was confirmed by the presence of two pseudomolecular ions *m/z* 326.2 ([M + H]^+^) and 348.2 ([M + Na]^+^). The pseudomolecular ion 326.2 ([M + H]^+^) was subsequently selected as a precursor for unambiguous HR-MS/MS identification *m/z*: 326.2327 (C_12_H_19_NO_4_, [M + H]^+^), 225.1819 (C_14_H_25_O_2_^+^), 183.1717 (C_12_H_23_O^+^), 102.0524 (C_4_H_8_NO_2_^+^), 74.0591 (C_3_H_8_NO^+^), 56.0492 (C_3_H_6_N^+^). All diagnostic adduct and fragment ions listed above were confirmed by analyzing the reference standard.

### 2.2. Expression of the AttM Lactonase Abolishes AHL Accumulation in *S. meliloti*

**Figure 2:**
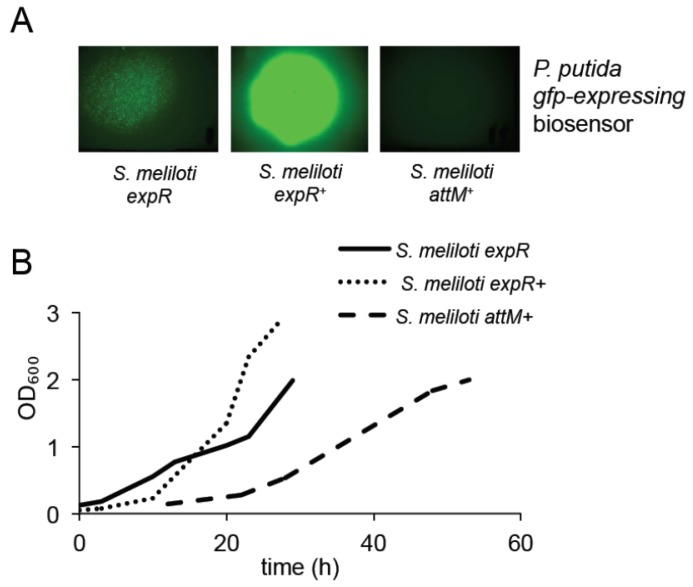
*S. meliloti* Rm2011 (*expR*) strain has lower, and *S. meliloti* Rm2011 (*attM^+^*) abolished AHL production in comparison to *S. meliloti* Rm2011 (*expR*^+^). All strains of *S. meliloti* were tested for the AHL production using the biosensor, *gfp*-expressing strain *P. putida* KS35 (**A**) AHL produced by *S. meliloti* strains were extracted with CHCl_3_ and applied onto a lawn of *P. putida* KS35 bacteria. The GFP signal was observed 2 h thereafter. *S. meliloti attM^+^* constitutively expresses the *attM* gene from *A. tumefaciens* coding for a lactonase. *S. meliloti expR* has a natural mutation in the AHL receptor gene *expR. S. meliloti expR*^+^ ectopically expresses *expR* gene; (**B**) Growth curves of *S. meliloti* strains with different AHL production.

Since the eventual aim of this study was to investigate the AHL-dependent interaction between bacteria and plants, we aimed at an AHL-negative strain of *S. meliloti*, which could be used as a control in our study. The *Agrobacterium tumefaciens attM* gene encoding a lactonase, an enzyme hydrolyzing the lactone ring of an AHL molecule, was chosen for expression in *S. meliloti* in order to achieve this purpose. After initial check of resistance (data not shown), the kanamycin-resistance conferring, broad host range plasmid pBBR2-attM was constructed and introduced into the *E. coli* S17-1 strain [14] used subsequently for conjugation with *S. meliloti* Rm2011. The resulting AHL-negative strain was tested for AHL accumulation using the bacterial biosensor strain *Pseudomonas putida* KS35 which expresses *lasB::gfp* in an AHL-dependent manner [14]. *S. meliloti* was grown until OD_600 nm_ = 1.3, and AHLs were extracted from culture filtrate. Extracted AHLs were redissolved in Me_2_CO (acetone) and applied onto lawns of biosensor bacteria, which recognize oxo-C14-HSL (Figure S1). *S. meliloti* Rm2011 (pBBR2-attM) called hereafter *attM^+^*, showed no detectable AHL accumulation (Figure 2A). In addition, we tested how the lack of AHLs influences the growth of rhizobia. The *S. meliloti attM* strain, unable to produce a detectable amount of oxo-C14-HSL, shows delayed growth if compared to the reference strains (*S. meliloti* Rm2011, ectopically expressing the *expR* gene from the pWBexpR plasmid, called hereafter *expR*^+^) [15], or even to the strain with a naturally occurring mutation in the AHL receptor-coding gene *expR* (*S. meliloti Rm2011 expR*) [12] producing reduced amount of oxo-C14-HSL when compared to the *S. meliloti expR*^+^ strain (Figure 2B).

### 2.3. Plants Impact on AHL Concentration

**Figure 3:**
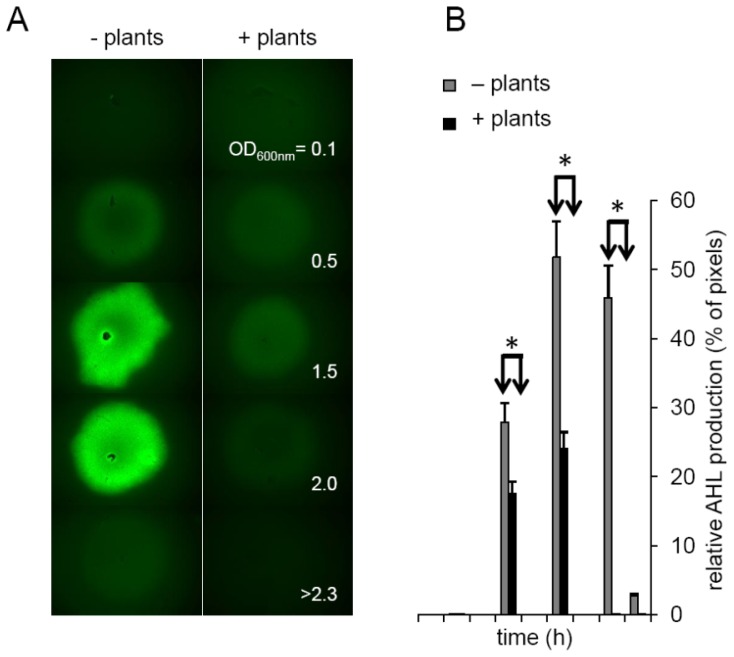
The presence of plants has a negative impact on the accumulation of AHLs produced by *S. meliloti.* (**A**) Accumulation of AHL produced by *S. meliloti expR*^+^ in culture with and without *Arabidopsis* plants was monitored using the bacterial biosensor strain *P. putida* KS35; (**B**) Quantification of the AHL production using a segmentation algorithm. Five photographs were taken per time point (see (**A**)) and tested for the ratio of GFP-positive pixels. The experiment was repeated five times. Error bars represent the S.D. between biological replicates. * represents *p* > 0.05 in Student’s *t*-test.

Next, we addressed the question whether plants influence the concentration of AHLs in their surroundings. To this end, we monitored the concentration of oxo-C14-HSL produced by *S. meliloti* Rm2011 *expR^+^* in bacterial culture in the presence or absence of *A. thaliana*. AHLs were monitored in filtrates from bacterial cultures grown at 22 °C until the OD_600 nm_ = 2.3. After extraction with CHCl_3_, the solvent was evaporated and the residue resuspended in Me_2_CO. AHLs were applied onto a lawn of the bacterial *gfp*-expressing biosensor strain *P. putida KS35*. The GFP signal was analyzed 2 h thereafter and quantified using the segmentation algorithm (Figure 3A,B). The concentration of oxo-C14-HSL reached a maximum at the late exponential phase (OD_600 nm_ = 1.5) and decreased during the stationary phase (at OD_600 nm_ > 2.3) (Figure 3A,B). However, if *Arabidopsis* plants were co-cultivated together with the *S. meliloti* bacteria, the AHL concentration peak was missing and the amount of AHLs was reduced (Figure 3A,B). This result suggests that plants have a negative impact on either the AHL production, on their stability or availability. Whether the effect originates from *Arabidopsis*-produced lactonases, inhibition of AHL production or adhesion of AHLs to plant cell walls is not yet known. The answer to this question requires further tests.

### 2.4. Oxo-C14-HSL Produced by *S. meliloti* Enhances Resistance of *Arabidopsis* Plants

**Figure 4:**
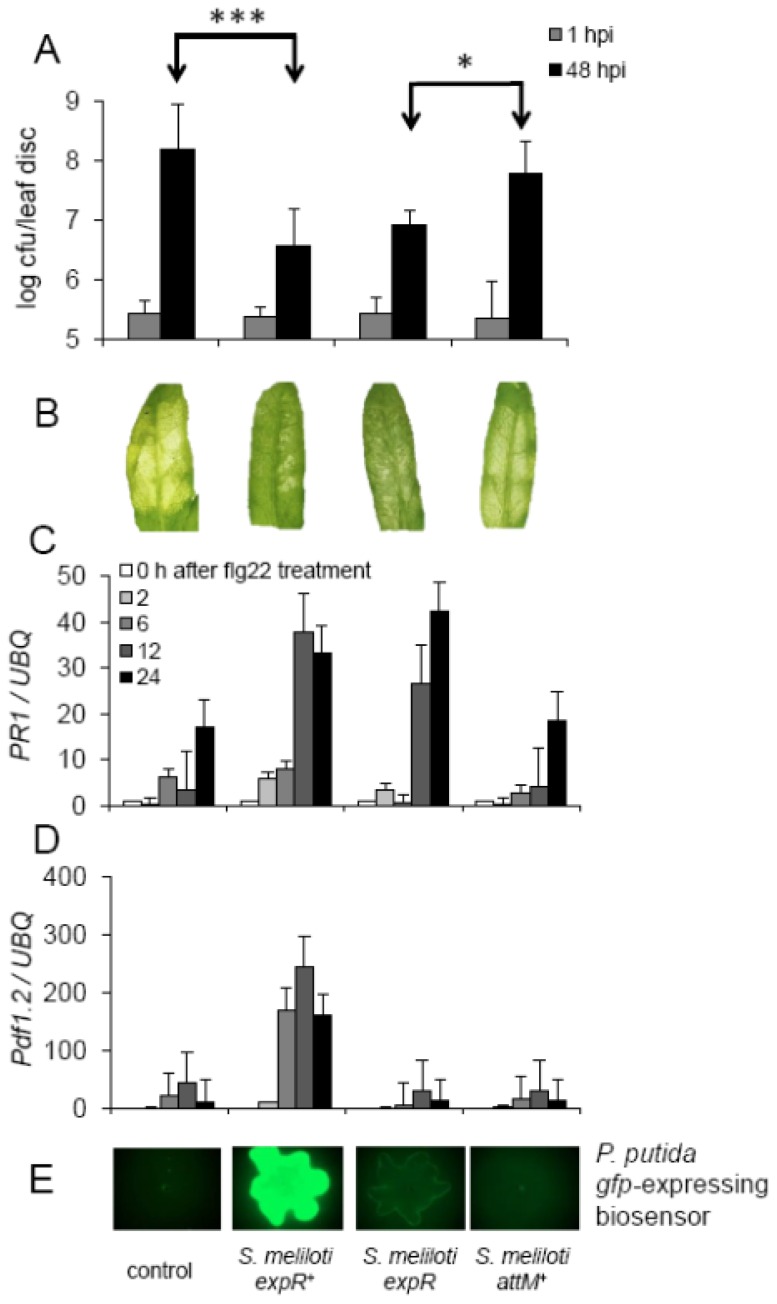
The *S. meliloti* strains producing oxo-C14-HSL enhance resistance in *Arabidopsis* plants. The enhancement in resistance towards the pathogenic bacteria in *Arabidopsis* is dependent on AHL. (**A**) Proliferation of the plant-pathogenic *P. syringae* DC3000 (*Pst*) on plants pretreated with MgCl_2_ (control) or different *S. meliloti* strains, producing different quantities of oxo-C14-HSL. *** *p* > 0.0005; * *p* > 0.05 in Student’s *t*-test. cfu; colony forming unit (**B**) Symptoms caused by infiltration with *Pst* bacteria into *Arabidopsis* leaves; (**C**) and (**D**) Expression of the defense related *Pathogenicity Related 1* (*PR1*) (**C**) and *Pdf1.2* (**D**) genes. Expression was normalized to the expression of the *At5g25760* gene (*UBQ)*. Total RNA was extracted from 2-week-old *Arabidopsis* seedlings pre-treated for 3 days with AHL extract from different *S. meliloti* cultures and challenged with 100 nM flg22 for hours as indicated. Graphs present a representative experiment from three independent replicates; (**E**) AHL production in *S. meliloti* strains used in the above experiments detected by the bacterial biosensor strain *P. putida* KS35.

Long-chain AHLs (e.g., oxo-C14-HSL) were shown to increase the resistance of barley and *Arabidopsis* plants towards hemibiotrophic pathogens; however, they show no effect on plant development [8,10]. To further substantiate our observation, we tested the impact of inoculation with *S. meliloti* strains producing oxo-C14-HSL on *Arabidopsis* resistance towards *Pseudomonas syringae* pv *tomato* (*Pst*) bacteria. We chose *Arabidopsis* rather than the native host of *S. meliloti*, *Medicago tuncatula*, in order to avoid effects related to nodulation and N_2_-fixation. The rhizosphere of soil-grown *Arabidopsis* plants was inoculated with different *S. meliloti* strains during a three-week period prior to the challenge. *Pst* bacteria were infiltrated into leaves and colony forming units (cfu) number was monitored one and 48 h after infiltration. In plants pre-treated with *S. meliloti expR*^+^, the proliferation of *Pst* bacteria was significantly slower than in control plants (Figure 4A). Moreover, the resistance-inducing effect seems to be AHL-dependent since pre-treatment with strains producing less or no oxo-C14-HSL (*S. meliloti expR* or *S. meliloti attM^+^*, respectively) has a reduced or no effect on resistance (Figure 4A).

The expression profiles of two pathogenesis-related (*PR*) genes: *PR1* and *Pdf1*.2, were in agreement with those results. Challenge with flg22 induced the expression of *PR1* 24 h after treatment in all plants (Figure 4C). However, in plants pre-treated with extract originating from *S. meliloti expR*^+^ or *S. meliloti expR*, the induction of *PR1* was drastically increased (Figure 4C). Similarly, expression of *Pdf1.2* was increased in plants pre-treated with extract from *S. meliloti expR*^+^, though not in control or plants pre-treated with extract from *S. meliloti* strains producing less or no AHLs, *S. meliloti expR* or *S. meliloti attM^+^* (Figure 4D). Together with the induced resistance towards *Pst*, these results suggest that the pre-treatment with the *S. meliloti* strains producing oxo-C14-HSL (Figure 4E) prime *Arabidopsis* plants for better defense responses. In such primed state plants are able to respond in a faster and/or stronger way to a secondary challenge (e.g., pathogen infection).

**Figure 5:**
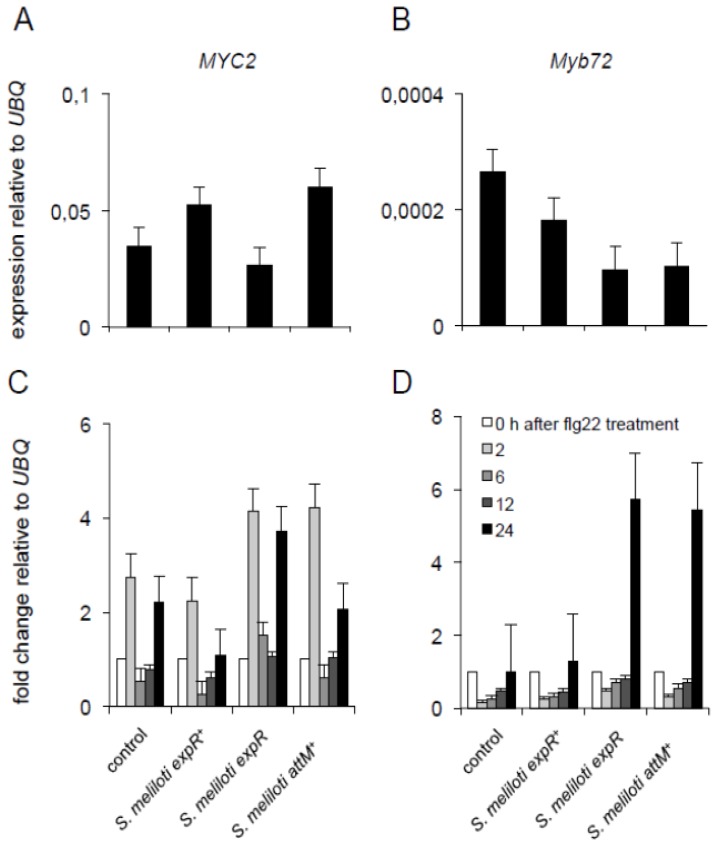
Expression of two Induced Systemic Resistance (ISR) related genes. Two-week-old *Arabidopsis* seedlings were pre-treated with AHL extract from different *S. meliloti* cultures, and challenged with 100 nM flg22. (**A**) and (**B**) Expression levels of *MYC2* (**A**) and *Myb72* (**B**) after 3 days of pre-treatment; (**C**) and (**D**) Expression levels after the secondary challenge with 100 nM flg22. All values were normalized to the expression of *UBQ* gene. Graphs present a representative experiment from three independent replicates.

Notably, this priming seems to be independent of the previously described priming mechanisms. The inoculation with AHL extracts from *S. meliloti expR*^+^ culture neither has an impact on the expression of the ISR associated *MYC2* and *Myb72* transcriptions factors (Figure 5A,B), nor on their expression of those genes after a secondary challenge with flg22 (Figure 5C,D) if compared to control plants. Nevertheless, we observed an increase in the expression of both *Myb72* and *MYC2* after the challenge with flg22, in plants pretreated with extracts from either *S. meliloti expR* or *S. meliloti attM^+^* (Figure 5C,D). These results suggest that the long-chain oxo-C14-HSL from *S. meliloti* interferes with the flg22-induced expression of both ISR associated genes, a phenomenon observed also for the treatment with pure oxo-C14-HSL [16]. The exact mechanism of this interaction is still not clear.

**Figure 6:**
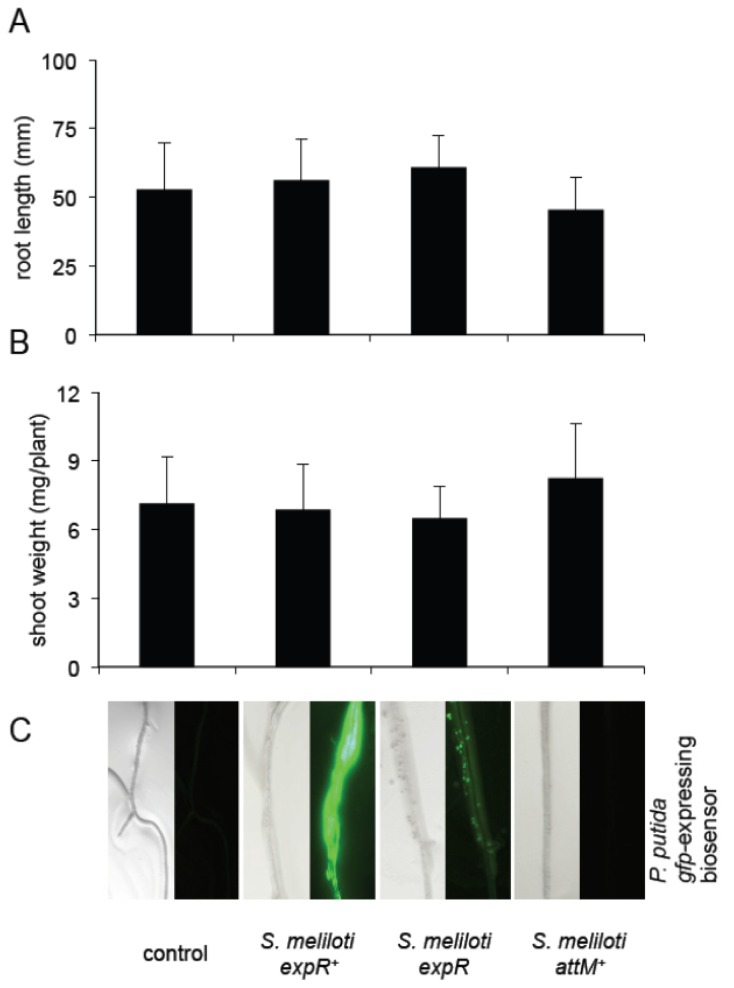
*S. meliloti* strains producing oxo-C14-HSL has no impact on the growth of *Arabidopsis* plants. (**A**) Root length of *Arabidopsis* plants co-inoculated with *S. meliloti* strains for 9 days; (**B**) Fresh weight of shoots of plants inoculated with *S. meliloti* strains during 15 days; (**C**) AHL production by *S. meliloti* strains used in the above experiments on *Arabidopsis* roots detected by the biosensor bacteria *P. putida* KS35. Student’s *t*-test revealed no differences between control and the different treatments, at *p* ≥ 0.05 in values presented in (**A**) and (**B**).

Next, we examined whether the inoculation with *S. meliloli* has an impact on *Arabidopsis* growth. To this end, we transferred one-week-old seedlings on vertical plates and inoculated them with *S. meliloti expR*^+^, *S. meliloti expR*, and *S. meliloti attM^+^*. The root tip positions were measured during 9 days after inoculation and pants weight was measured after 15 days. We observed no differences between the control plants and any of the treatments (Figure 6), suggesting that neither the *S. meliloti* nor its long-chain AHL has an impact on *Arabidopsis* growth.

### 2.5. Oxo-C8-HSL Produced by *R. etli* Has only Moderate Impact on *Arabidopsis* Growth

**Figure 7:**
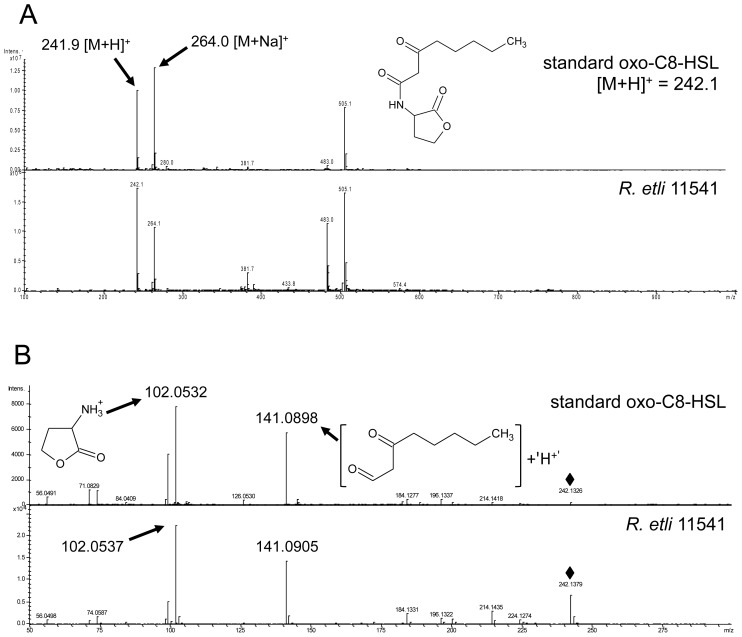
Verification of AHLs production in *Rhizobium etli* 11541. HPLC samples were screened in the positive ion mode, and a scan from *m/z* 50 to 500 was performed. (**A**) The MS of standard oxo-C8-HSL standard (*m*/*z* = 242.1 [M + H]^+^) shows a pattern similar to AHLs extracted from the *R. etli* culture; (**B**) Further analysis by MS/MS confirmed the identity of AHL extracted from the *R. etli* culture with the standard oxo-C8-HSL. The diagnostic fragment ions *m*/*z* 102.0532 and 102.0537 are indicative for the presence of the lactone ring. The highlighted *m*/*z* values represent diagnostic fragment ions. Diamonds mark the pseudomolecular ions used for MS/MS analysis.

Previous reports on short-chain (C6-HSL and oxo-C8-HSL) AHLs influence on plants suggested that those molecules possess a growth-promoting effect [6,8,17]. Therefore, in the second part of this study, we addressed the question whether the presence of rhizobia producing short-chain AHLs would have a positive impact on plants. *R. etli* was previously shown to produce oxo-C8-HSL and was chosen in this study [18]. In the first step, we verified the nature of the AHLs produced by *R. etli* 11541 under our conditions. A peak overlapping with oxo-C8-HSL was identified by LC-MS. The presence of oxo-C8-HSL was subsequently confirmed by MS/MS (Figure 7). This result is in line with the previous reports on AHL production by *R. etli*. The identity of oxo-C8-HSL was unambiguously confirmed by the presence of the following adduct ions *m/z*: 241.9 ([M + H]^+^), 264.0 ([M + Na]^+^), 483.0 ([2M + H]^+^) and 505.1 ([2M + Na]^+^). The pseudomolecular ion 241.9 ([M + H]^+^) was subsequently selected as a precursor ion for unambiguous HR-MS/MS identification *m/z*: 242.1326 (C_12_H_19_NO_4_, [M + H]^+^), 141.0898 (C_8_H_13_O_2_^+^), 102.0532 (C_4_H_8_NO_2_^+^), 74.0587 (C_3_H_8_NO^+^), 71.0829 (C_5_H_11_^+^) and 56.0491 (C_3_H_6_N^+^). All diagnostic adduct and fragment ions listed above were confirmed by analyzing the reference standard. In addition, in analogy to *S. meliloti*, we constructed two AHL-negative strains in which the accumulation of oxo-C8-HSL was abolished due to the expression of the *attM* lactonase gene of *A. tumefaciens* using two different pBBR-variants conferring kanamycin and gentamicin resistance, respectively (Figure S2).

**Figure 8:**
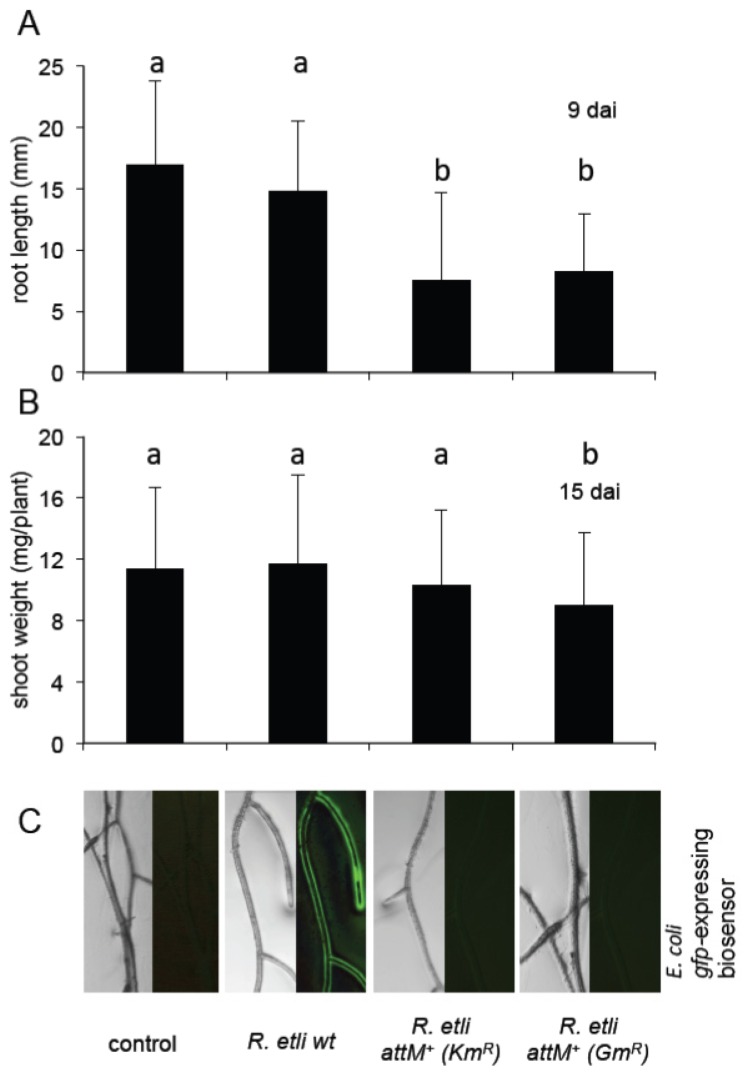
*R. etli* producing oxo-C8-HSL has only moderate impact on *Arabidopsis* growth. (**A**) Root length of *Arabidopsis* plants co-inoculated with the indicated *R. etli* strains for 9 days; (**B**) Fresh weight of shoots of plants inoculated with *R. etli* strains during 15 days; (**C**) AHL production by *R. etli* strains used in the above experiments on *Arabidopsis* roots detected by the biomarker bacteria *E. coli*. Letters indicate statistical differences in Student’s *t*-test at *p* ≥ 0.005 (**A**) or *p* ≥ 0.05 (**B**), dai; day after inoculation.

In the next step, we assessed whether the oxo-C8-HSL produced by *R. etli* (Figure 7) influences the growth of *Arabidopsis*. In line with the tests with *S. meliloti*, we chose *Arabidopsis* rather than the native host (*Phaseolus vulgaris*) in order to avoid the nodulation and N_2_-fixation related phenotype, which could mask the AHL effect. Besides, *R. etli* had been already reported to be a plant growth promoting rhizobacterium (PGPR) on non-legume plants [19]. To verify the effect of *R. etli*-originated oxo-C8-HSL, one-week-old *Arabidopsis* seedlings were transferred to a vertical growth system (square Petri dishes with half MS medium and no sucrose supply) and inoculated with *R. etli* wild type (wt), *R. etli attM^+^* (*Km^R^*), and *R. etli attM^+^* (*Gm^R^*) (OD_600 nm_ < 0.1), 10 mM MgCl_2_ solution was used as a control. The root tip position was measured every 3rd day. After 9 days we observed no differences between plants inoculated with *R. etli* wt and those treated with MgCl_2_ solution (Figure 8A). However, plants inoculated with both lactonase-expressing strains: *R. etli attM^+^* (*Km^R^*) and *R. etli attM^+^* (*Gm^R^*) showed significantly shorter roots (Figure 8A). Likewise, the shoot weight measured 15 days after inoculation was similar between controls and *R. etli* wt treated plants (Figure 8B). In contrast, plants inoculated with *R. etli attM^+^* (*Gm^R^*) had significantly lighter rosettes (Figure 8B). We hypothesize that the oxo-C8-HSL produced by the wild type *R. etli* strain (Figure 8C) balances the otherwise negative impact of *R. etli* colonization on *Arabidopsis* growth.

A possible explanation why *Arabidopsis* reacts to *R. etli* colonization with a growth inhibition could be an induced defense mechanism. To prove this assumption we tested the resistance towards the hemi-biotrophic *Pst* bacteria. Two different infection methods with *Pst* were chosen: infiltration and spray-inoculation. Soil-grown *Arabidopsis Col*-0 wild type plants were watered with *R. etli* wt, *R. etli attM^+^* (*Km^R^*) and *R. etli attM^+^* (*Gm^R^*) (OD < 0.1) suspension during three weeks prior challenge with *Pst* bacteria. Cfu of *Pst* were analyzed one and 48 h after infiltration in leaf discs. We observed no differences in the resistance or susceptibility towards *Pst* (Figure S3A). Similarly, the spray-inoculation of hydroponically grown *Arabidopsis* with *Pst* revealed no difference in the resistance towards this pathogen (Figure S3B), suggesting that *R. etli* has no influence on the resistance towards *P. syringae* in *Arabidopsis* plants.

## 3. Discussion

In this report, we investigated the impact of bacteria-originated AHLs at defense responses and growth of *A. thaliana*. Both bacterial species used in this study are well-studied rhizobia entering close symbiosis with legumes. On the one hand, we chose *S. meliloti*, which produces several AHLs, including oxo-C14-HSL [11,20]. As recently shown, long-chain AHLs induce resistance in *Arabidopsis* and barley plants [8,10]. Here, we show the positive effect of a strain producing high amounts of oxo-C14-HSL (*S. meliloti expR^+^*) on plant resistance. As expected, this bacterial strain has no influence on plant growth. We propose an AHL-induced priming as the mechanism of increased resistance. Interestingly the AHL-induced priming seems to be different from the priming induced by other PGPR, as shown by the expression of *MYC2* and *Myb72* transcription factors [21,22]. Notably, the AHL synthase (*sinI*) and AHL receptor (*expR*) expressed in free-living rhizobia are repressed in bacteria living in a symbiosis with the native host *M. truncatula* [23]. We therefore hypothesize, that in case of nodule-living bacteria the AHL-priming effect is missing. On the other hand, we confirmed our previous findings that short-chain AHLs, like oxo-C8-HSL from *R. etli*, have no resistance-inducing activity. Unfortunately, the expected positive effect of bacterial oxo-C8-HSL on plant growth is only very moderate; indicating that the interaction between plant and rhizobia depends on more signals than AHLs. Taken together this study provides new arguments supporting the observation that plants respond to bacterial AHLs and that this response depends on the length of the AHLs.

### 3.1. AHL Production in *S. meliloti* and *R. etli*

The impact of bacterial AHLs on plants was postulated in several independent studies [3,6,10,17,24]. However, only recent results indicated that different molecules might have different influence on plant hosts [8]. Others and our studies suggested that the short-chain AHLs increase the growth of *Arabidopsis* plants [6–8,17], whereas the long-chain AHLs reinforce the plant resistance against biotrophic and hemibiotrophic pathogens [10]. Therefore, the fact that the bacteria chosen in this study produce either long or short-chain AHLs allowed to verify our previous hypothesis on AHLs bi-functionality in respect to plant reactions [25]. Very remarkable was the specificity in the AHL type. Both bacterial species, *S. meliloti* and *R. etli*, were already examined in this regard, the AHLs identified in this report were previously reported to be within the palette of homoserine lactones produced by the respective bacterial species [11,18,20].

### 3.2. Systemic Induction of *Arabidopsis* Resistance by *S. meliloti* Treatment

Previous studies have shown that *S. meliloti* produces different long-chain AHLs including 3-oxo-C14-, C16-, 3-oxo-C16-, C16:1-, and 3-oxo-C16:1-HSL [26,27]. In this study, we confirmed the presence of oxo-C14-HSL in *S. meliloti* culture. Because of the previously observed, positive impact of oxo-C14-HSL treatment on *Arabidopsis* and barley resistance [10], we expected that *S. meliloti* strains producing oxo-C14-HSL will have positive effect on *Arabidopsis* resistance. Indeed, co-cultivation of *Arabidopsis* plants with the *S. meliloti expR*^+^ strain, which produces high amounts of oxo-C14-HSL, significantly enhances the resistance towards the pathogenic *Pst* bacteria. In addition, also the *S. meliloti expR*, which produces lower amount of oxo-C14-HSL, has a similar, positive impact on plant resistance. In the same context, plants pretreated with the lactonase-expressing *S. meliloti attM^+^* strain showed increased *Pst* proliferation when compared to plants pretreated with the oxo-C14-HSL- producing *S. meliloti* strains. These results indicate an involvement of the AHL in the resistance conferred by *S. meliloti*. Similar results have been obtained by Schuhegger *et al*. [3], these authors reported the induction of resistance against the fungal leaf pathogen *Alternaria alternata*, in tomato plants pretreated with *Serratia liquefaciens* MG1. Furthermore, they indicated that the AHL-negative mutant of *S. liquefaciens* MG44 was less effective in induction of resistance against *A. alternata* [3]. Intriguing is the fact that although the major AHL produced by *S. liquefaciens* is the short-chain C6-HSL [28], it appears to confer resistance to a necrotrophic pathogen. Unfortunately, we were not able to verify this effect with pure AHL or the oxo-C8-HSL-producing *R. etli* strain (data not shown), leaving the question on the impact of short-chain AHLs on resistance towards necrotrophic pathogens open. Interesting to note are the different capabilities of moving throughout a plant of short and long-chain AHLs, while the short-chain C6-HSL was found in shoot of *Arabidopsis* when applied to root, the long-chain AHL oxo-C14-HSL was not [10]. In addition, also the substitutions in the lipid chain play a role in induced resistance [10], indicating that besides the specific response to a given AHL, plants possess also systemic signal, which regulates the AHL-priming.

### 3.3. Growth Inducing Capacities

Short-chain AHL was shown to promote growth in *Arabidopsis* plants [6–8,17]. Depending on the exact length, the possible effect may include also alteration in root hair morphology and thickening of roots [17]. The short-chain oxo-C8-HSL produced by *R. etli* (this work and [18]) encouraged us to test whether the inoculation with AHL-producing bacteria has effect similar to the pure molecule. As concluded above*,* despite the fact that it has been already reported to be a plant growth promoting rhizobacterium (PGPR) on the non-legume tomato and pepper plants [19], *R. etli* has a negative effect on *Arabidopsis* growth. However, this negative impact is possibly contra-balanced with the positive effect of oxo-C8-HSL produced by this bacterium. Such compensation phenomenon could be the explanation for the reduced growth of plants pretreated with the lactonase-expressing strains (*R. etli attM^+^*, *Km^R^* and *Gm^R^*), in comparison to growth at nearly control level of plants pretreated with the wild type *R. etli* producing short-chain AHL. These results appear to be in line with reports that only a few rhizobacteria are known to be naturally associated with *Arabidopsis* roots, and only few showed positive growth effects [29]. As expected, treatment with *S. meliloti* has no influence on the growth of *Arabidopsis.* Neither the *S. meliloti expR^+^*, producing high levels of AHLs, nor the AHL-negative (*S. meliloti attM^+^*) strain influenced the growth rate of *Arabidopsis*.

### 3.4. Impact of Plant on AHL Production

Several previous reports demonstrated the effect of AHLs on biofilm formation in bacterial communities. Recently, a model assuming the role of autoinducers in promotion of highly adaptable, spatial heterogeneity in populations was suggested [30]. Notably, the presence of plants diminished the concentration of AHL in bacterial culture, without major effect on the bacterial proliferation (Figure S4). While some explanations are possible: adhesion to root surface or a residual lactonase activity of the five phosphogluconolactonases encoded on *Arabidopsis* genome, the most probable is however a quorum quenching (QQ) activity of root exudates. Such activity was already reported for several higher plants and algae [31–36]. Between many unidentified QS-mimicking and QQ molecules in root exudates, halogenated furanones, *L*-canavanine and flavan-3-ol catechin have been identified as plant-originated inhibitors of QS [34–36]. Especially the halogenated furanones from the red alga *Delisea pulchra* act directly at the AHL reception site by facilitating the degradation of the AHL-LuxR complex and therefore inhibiting of QS-depending processes [34]. Whether higher plants secret similar compounds was not yet reported. A screening for QS active substances in the native host of *S. meliloti*, *Medicago truncatula*, revealed 15 to 20 substances, which either activate or inhibit QS [32]. A similar study identified QS-interfering activities in exudates from rice and bean plants [33]; however, the nature of those molecules remains undetermined. Whether *Arabidopsis* exudates compound(s) with similar activity is probable, though not yet proven. Very intriguing is also the possibility that plant-originated molecules influence not only the perception or stability of QS molecules, but also the synthesis.

Taken together this report demonstrates the dependence of the plant reaction to rhizobia on the type of AHL, produced by those bacteria. The AHL-priming effect detected in plants pre-treated with oxo-C14-HSL producing *S. meliloti* strains shows that the complex interaction between bacteria and plants goes further than the perception of MAMPs and effectors or even NOD factors. Additional experiments shall clarify the mechanisms of AHL-priming and its potential in agriculture.

## 4. Experimental Section

### 4.1. Plant

Wild type *Arabidopsis thaliana Col-0* (ecotype Columbia) was obtained from The Nottingham *Arabidopsis* Stock Center (NASC), NASC ID: N60000. Plants were either grown on soil under short-day conditions (8/16 h light regime, at 21 °C) or in sterile condition on half MS medium supplemented with 0.8% agar at 21 °C. For systemic approach, plants were grown in sterile hydroponics culture using half MS medium without sucrose, assuring the separation between roots and shoot parts.

### 4.2. Bacterial Strains and Growth Conditions

*R. etli* 11541 wild type was obtained from DSMZ (German Collection of Microorganisms and Cell Cultures) in Braunschweig, Germany. *S. meliloti* Rm2011, an *expR* mutant (*expR*), was obtained from A. Becker. *S. meliloti* Rm2011 *expR^+^* containing the pWBexpR plasmid was obtained from M. McIntosh. All used *S. meliloti* strains are resistant to streptomycin (250 μg/μL).

*R. etli* (pBBR2-attM*), R. etli* (pBBR5-attM) and *S. meliloti* (pBBR2-attM) carrying the lactonase gene *attM* from *Agrobacterium tumefaciens* were obtained by conjugation with *Escherichia coli* S17-1 carrying *attM* on pBBR1MCS-2 (conferring kanamycin resistance, *Km^R^*) or pBBR1MCS-5 (conferring gentamicin resistance, *Gm^R^*) plasmids [37].

Rhizobia were grown in 5 mL culture in a 50 mL Erlenmeyer flask under 100 rpm constant agitation at 21 °C in TY medium [13]. The OD was measured every 4 h using 50 μL, we performed dilutions for ODs above 0.8.

*E. coli* MT102 (pJBA89) resistant to ampicillin 100 μg/mL [38], and *P. putida* KS35, resistant to gentamicin (20 μg/mL) and kanamycin (50 μg/mL) [39] were used for AHLs detection.

### 4.3. AHL Detection and Quantification

#### 4.3.1. Chemicals

All solvents used for LC/MS analyses, acetonitrile (MeCN, 99.9%), and formic acid (FA, 98%), were of LC/MS grade from Sigma-Aldrich (Steinheim, Germany). Water was purified by a Merck-Millipore Milli-Q Synthesis A10 system (Merck-Millipore, Schwalbach/Ts., Germany).

#### 4.3.2. HPLC/MS-MS

In order to identify the type of AHLs produced by *R. etli* and *S. meliloti,* bacteria were grown on TY media (OD_600 nm_ ≈ 0.8) with the respective antibiotics in a large scale (80 mL). The cultures were centrifuged to remove the bacterial cells, and 5 mL CHCl_3_ was added for extraction of AHLs. AHL extract were evaporated and redissolved in 80% MeCN, vortexed, placed for 15 min in an ultrasonic bath (Sonorex, Bandelin, Berlin, Germany) and centrifuged for 15 min at 21,000× *g* (Mikro 220R, Hettich, Tuttlingen, Germany). For LC/MS analysis and fractionation, two mass spectrometers, both from Bruker Daltonics (Bremen, Germany), were used. Both instruments were controlled by the HyStar software (version 3.2, SR 2, Bruker Daltonics, Bremen, Germany, 2012). The instruments were equipped with an orthogonal ESI source.

For separation and fractionation of different AHL-types, an amaZon ETD Ion-Trap MS, coupled to a Dionex UltiMate 3000 HPLC (Dionex, Idstein, Germany), has been used. Samples were separated on an Acclaim 120 C18, 3 μm, 120 Å, 4.6 × 150 mm column (Dionex, Idstein, Germany) at a flow rate of 1 mL/min and at 35 °C. Eluent A consisted of H_2_O + 0.1% FA, eluent B of 80% MeCN + 0.1% FA. Samples of 150 μL were injected. A linear gradient of 5%–100% B in 40 min was used. The source parameters were adjusted as follows: capillary voltage 4500 V; end plate offset 500 V; nebulizer 1 bar, dry gas 8 L/min with a dry temperature of 200 °C. The mass accuracy of the low-resolution system is ±0.2 Da. Before injection into the Ion-Trap MS, the flow was split: one third was used for monitoring of the different AHLs in the positive ion mode using a full scan from *m*/*z* 100 to 2000. Two thirds were used to fractionate the samples. Over a time period of 80 min, fractions were collected every 30 s. These fractions were evaporated using ultra-speed vacuum centrifugation and redissolved in 80% Me_2_CO (acetone) in order to be suitable for the GFP biosensor bacteria assay described below. Only bioactive fractions were used for subsequent identification.

A high-resolution microTOF-Q II mass spectrometer was used for identification of bioactive secondary metabolites. For direct infusion experiments, samples were applied via syringe pump at a flow rate of 180 μL/h. The source parameters were adjusted as follows: capillary 4500 V, end plate offset 500 V, nebulizer 0.4 Bar, dry gas 4 L/min, dry temprature 180 °C. The ion optics were adjusted as follows: funnel 1 RF 200 Vpp, funnel 2 RF 200 Vpp, ISCID energy 0 eV, hexapole RF 100 Vpp, ion energy 3 eV, collision RF 180 Vpp, collision energy 8 eV, transfer time 80 μs, and pre puls storage 7 μs. The mass accuracy of the high-resolution system is ±10 mDa. Positive-mode MS/MS experiments were performed in the *m*/*z* range from 50 to 500. Precursor ions were fragmented at collision energy of 20–30 eV. Data interpretation was performed using the DataAnalysis software (version 4.0 SP 5, Bruker Daltonic, Bremen, Germany, 2012). Standards of C6-, oxo-C8-, oxo-C10-, oxo-C12-, and oxo-C14-HSLs from Sigma-Aldrich were used as reference standards in both systems.

#### 4.3.3. AHL Detection Using Biosensor Bacteria

AHLs were extracted either from the liquid medium where plants were growing (as described in plant growth conditions) or from bacterial culture. All extractions were performed by adding CHCl_3_, centrifugation, and discarding the aqueous phase. CHCl_3_ was evaporated using ultra-speed vacuum centrifugation and the residue was redissolved in Me_2_CO. In order to detect AHLs production, two reporter bacteria were used: *Escherichia coli* MT102 (pJBA89), which is able to detect all side chain lengths of AHLs, and *P. putida* KS35 that can perceive only long-chain AHLs. These *gfp*-expressing biosensor bacteria were grown on LB medium with the corresponding antibiotics over night at 21 °C and plated on LB agar plates for one more night. Five microliters of extracted and redissolved AHLs were dropped on the plates containing a lawn of the reporter bacteria and detection of GFP expression was done after 2 h by fluorescence binocular microscope.

### 4.4. Segmentation Algorithm

In order to objectively analyze the fluorescence response of the reporter bacteria a full automatic algorithm has been developed which returns the relative AHL production as a percentage of all pixels in the microscopic images. The key idea is to segment the image into two disjoint region sets. The first one will represent the fluorescing bacteria (foreground) and the second one the non-fluorescing parts (background) in the image. Given this segmentation, the quotient between the size of the foreground region set and the whole image size is the relative AHL production. The segmentation algorithm is an adaptation of the work presented in [40]. Here, an energy functional is minimized, whose minimum is a binary image representing the segmentation result. As input for this process, a user defines on an arbitrary image the foreground and background region. The algorithm trains from this a model how pixels and its neighbors appear for a given region. This model was used to evaluate all microscope images.

### 4.5. Growth Promotion Assay

One-week-old *Arabidopsis* seedlings were transferred into half MS square plates without sucrose. The root tip position was marked and 10 μL of bacterial cultures (OD_600 nm_ < 0.1) were dropped on the root tip. Root length was measured manually every 3rd day. The roots and leaves weight was measured at the end of the experiment. The bacterial treatments included the indicated bacterial strains as well as MgCl_2_ used as a control.

### 4.6. Pathogenicity Assays

Two methods for *Pseudomonas syringae* pv. *tomato* DC3000 (*Pst*) quantification were tested. Roots of 6-week-old *Arabidopsis thaliana Col-0* grown in a sterile systemic system were pretreated with rhizobia bacteria. After 3 days, shoots were spray-inoculated with *Pst* at OD_600 nm_ = 0.1 in 10 mM MgSO_4_, 0.02% Silwet77. Leaves were harvested after 1 h and 96 h post inoculation (hpi) and homogenized. A serial of dilutions with (10 mM MgSO_4_) from the homogenized plant materials was made and dropped on King’s B medium. The colony forming units (cfu) of *Pst* were counted 2 days later in order to assess the proliferation of *Pst*. In the second approach *Arabidopsis* plants were grown on soil for four weeks as described under plant growth conditions. Plants have been pretreated 3 times with (1–4 mL) of bacterial cultures grown until exponential phase. Three days after the last treatment, leaves where infiltrated with *Pst* (OD _600 nm_ = 0.01) diluted in 10 mM MgSO_4_. A biopsy punch was used to prepare leaf discs with 5 mm diameter at 1 hpi and 48 hpi. The leaf discs have been homogenized, diluted and used as described in the first pathogenicity assay to count the cfu of *Pst*.

### 4.7. Gene Expression Analysis

We used quantitative RT-PCR for all gene expression analyses. Total RNA was extracted with 1 mL Trizol (PeqLab, Erlangen, Germany) according to manufactures’ protocol. Two μg of total RNA was used for DNaseI digestion. cDNA synthesis was followed according to the qScript cDNA Synthesis Kit from Quanta BioScience Inc. (Gaithersburg, MD, USA). In order to check the efficiency of the reverse transcription, a semi-quantitative amplification of the *actin2* transcript (cycles: 28, annealing temp. 55 °C) was performed. A positive genomic DNA control was included to verify the purity of cDNA samples from a genomic DNA. Fifty nanogram cDNA was used for quantitative RT-PCR (Applied Biosystems 7500 real-time PCR system, Foster City, CA, USA). Annealing temperature was set to 60 °C in every qRT-PCR and the cycle number was set to 42. All expression values were normalized to the expression of *UBQ* gene and to the 0 hours post infection (hpi) values. Quantitative RT-PCR was done using specific primers: *At5g25760* (*UBQ*) fwd.: GCTTGGAGTCCTGCTTGGACG rev.: CGCAGTTAAGAGGACTGTCCGGC; *PR1* fwd.: GGAGCGGTAGGCGTAGGTCCC, rev.: CCCACGAGGATCATAGTTGC; *Pdf1*.2 frw.: GTTTGCTTCCATCATCACC, rev.: GGGACGTAACAGATACACTTG; *Myb72* frw.: TCATGATCTGCTTTTGTGCTTTG, rev.: ACGAGATCAAAAACGTGTGGAAC; *MYC2* frw.: TCATGATCTGCTTTTGTGCTTTG, rev.: ACGAGATCAAAAACGTGTGGAAC.

## 5. Conclusions

The interaction between root-associated bacteria and plants encompasses many levels. In addition to the well-studied symbiotic interaction between members from the Fabaceae family and rhizobia and the diverse pathogenic bacteria and their hosts, we present here the impact of bacterial quorum sensing molecules on plants resistance. In agreement with observations done on the impact of pure molecules from the acyl-homoserine lactones group on plants, we show here that also bacteria-originated molecules have similar qualities. The inoculation with long-chain AHL (oxo-C14-HSL) producing *S. meliloti* strains enhances the resistance of *Arabidopsis* plants, while inoculation with a strain unable to accumulate oxo-C14-HSL did not have this effect. Moreover, the resistance enhancement depends on the length of the acyl side chain because the inoculation with short-chain (oxo-C8-HSL) producing *R. etli* had no effect on the defense mechanism. These results indicate that plant perceive the AHL in diversified manner. In addition, the AHL-producing bacteria could hold a potential for future agricultural applications.

## Supplementary Information

Figure S1 Detection and quantification of standard AHLs using the bacterial biosensor strains *P. putida* KS35 and *E. coli E. coli* MT102 (pJBA89). (**a**) The GFP-based system using the *P. putida* KS35 and *E. coli* MT102 (pJBA89) strains. AHLs were purchased from Sigma-Aldrich, resuspended in Me_2_CO at concentrations as indicated and 5 μL of the solution were applied onto bacterial lawns; (**b**) Quantification of the GFP photographs using the segmentation algorithm [40].

Figure S2 Production of AHL in *R. etli s*trains used in this work. Different strains of *R. etli* were tested for AHL production using the bacterial biosensor *E. coli* MT102 (pJBA89) (**A**) AHLs produced by the indicated *R. etli* strains were extracted with CHCl_3_ and applied onto a lawn of the *E. coli biosensor*. The GFP signal was observed 2 h thereafter. *R. etli attM^+^(Km^R^)* and *R. etli attM^+^(Gm^R^*) are strains expressing the *attM* gene from *A. tumefaciens* coding for a lactonase; (**B**) Growth curves of the *R. etli* strains. Bacteria were grown in 5 mL culture with constant agitation (100 rpm) at 21 °C. OD_600 nm_ was measured every 4 h.

Figure S3 *R. etli* strains have no impact on *Arabidopsis* resistance towards *P. syringae* bacteria. Proliferation of the plant pathogenic *P. syringae* DC3000 (*Pst*) on plants pretreated with MgCl_2_ (control) or different *R. etli* strains, producing different quantities of oxo-C8-HSL. (**A**) *Pst* bacteria were infiltrated into *Arabidopsis* leaves. OD_600 nm_ = 0.01; (**B**) *Arabidopsis* plants were spray-inoculated with *Pst* bacterial solution, OD_600 nm_ = 0.1; (**C**) AHL production in *R. etli* strains used in the above experiments detected by the bacterial biosensor strain of *E. coli*. Student’s *t*-test revealed no differences between the treatments at *p* ≥ 0.05, as indicated by the letters in (**A**) and (**B**).

Figure S4 Growth of *S. meliloti* in the presence of *Arabidopsis* plants. Proliferation of *S. melioti* measured over the period of 2 days in medium with or without *Arabidopsis* seedlings.
