# Supplementary figures and images for: Homoserine Lactones Influence the Reaction of Plants to Rhizobia
Source: Int J Mol Sci. 2013 Aug 20;14(8):17122–46. doi: 10.3390/ijms140817122 (PMC3759955; doi:10.3390/ijms140817122)

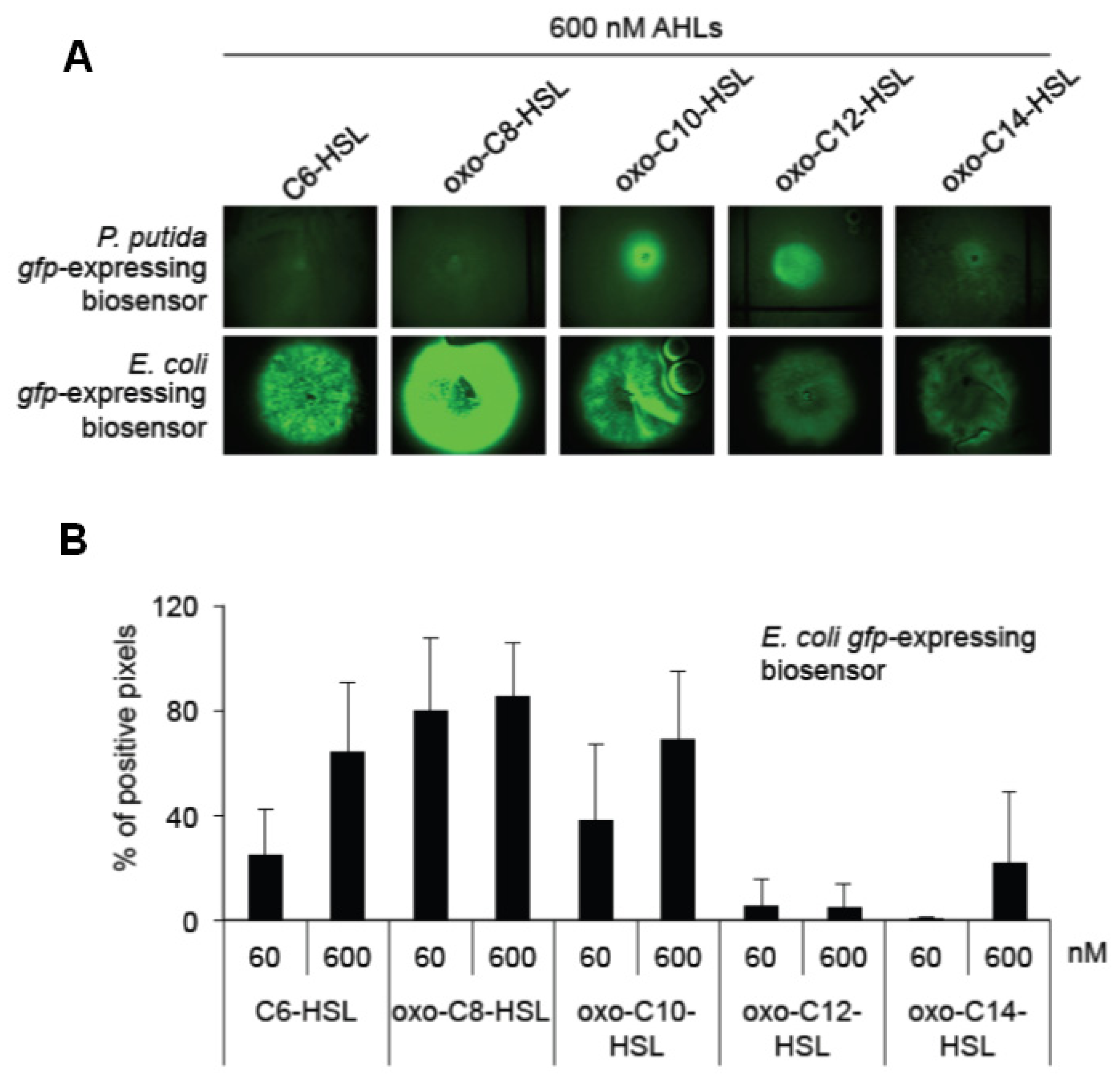

Supplement: Figure S1 — Detection and quantification of standard AHLs using the bacterial biosensor strains P. putida KS35 and E. coli E. coli MT102 (pJBA89). (a) The GFP-based system using the P. putida KS35 and E. coli MT102 (pJBA89) strains. AHLs were purchased from Sigma-Aldrich, resuspended in Me2CO at concentrations as indicated and 5 μL of the solution were applied onto bacterial lawns; (b) Quantification of the GFP photographs using the segmentation algorithm [40]. [file ijms-14-17122s1.tif]

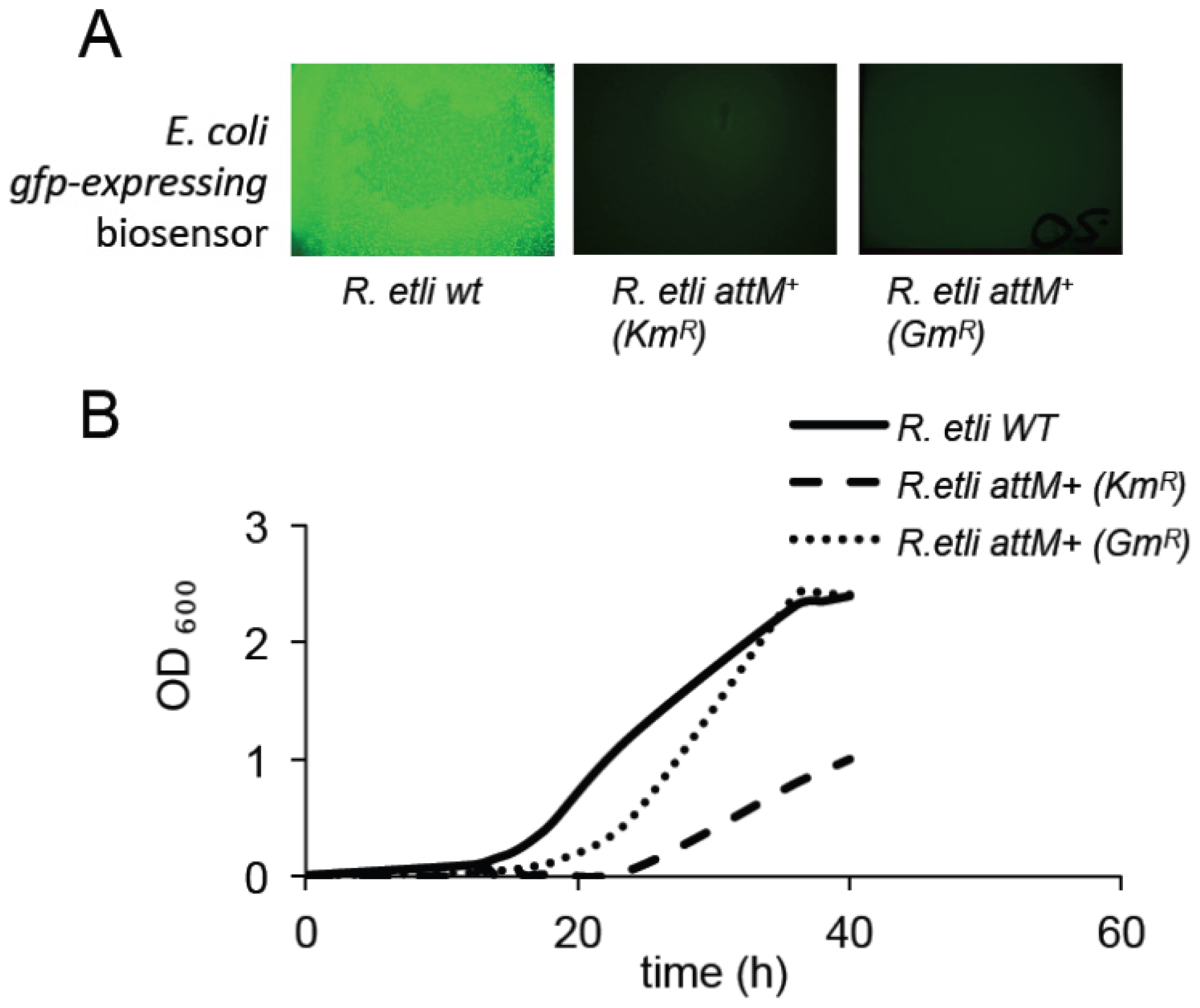

Supplement: Figure S2 — Production of AHL in R. etli strains used in this work. Different strains of R. etli were tested for AHL production using the bacterial biosensor E. coli MT102 (pJBA89) (A) AHLs produced by the indicated R. etli strains were extracted with CHCl3 and applied onto a lawn of the E. coli biosensor. The GFP signal was observed 2 h thereafter. R. etli attM+(KmR) and R. etli attM+(GmR) are strains expressing the attM gene from A. tumefaciens coding for a lactonase; (B) Growth curves of the R. etli strains. Bacteria were grown in 5 mL culture with constant agitation (100 rpm) at 21 °C. OD600 nm was measured every 4 h. [file ijms-14-17122s2.tif]

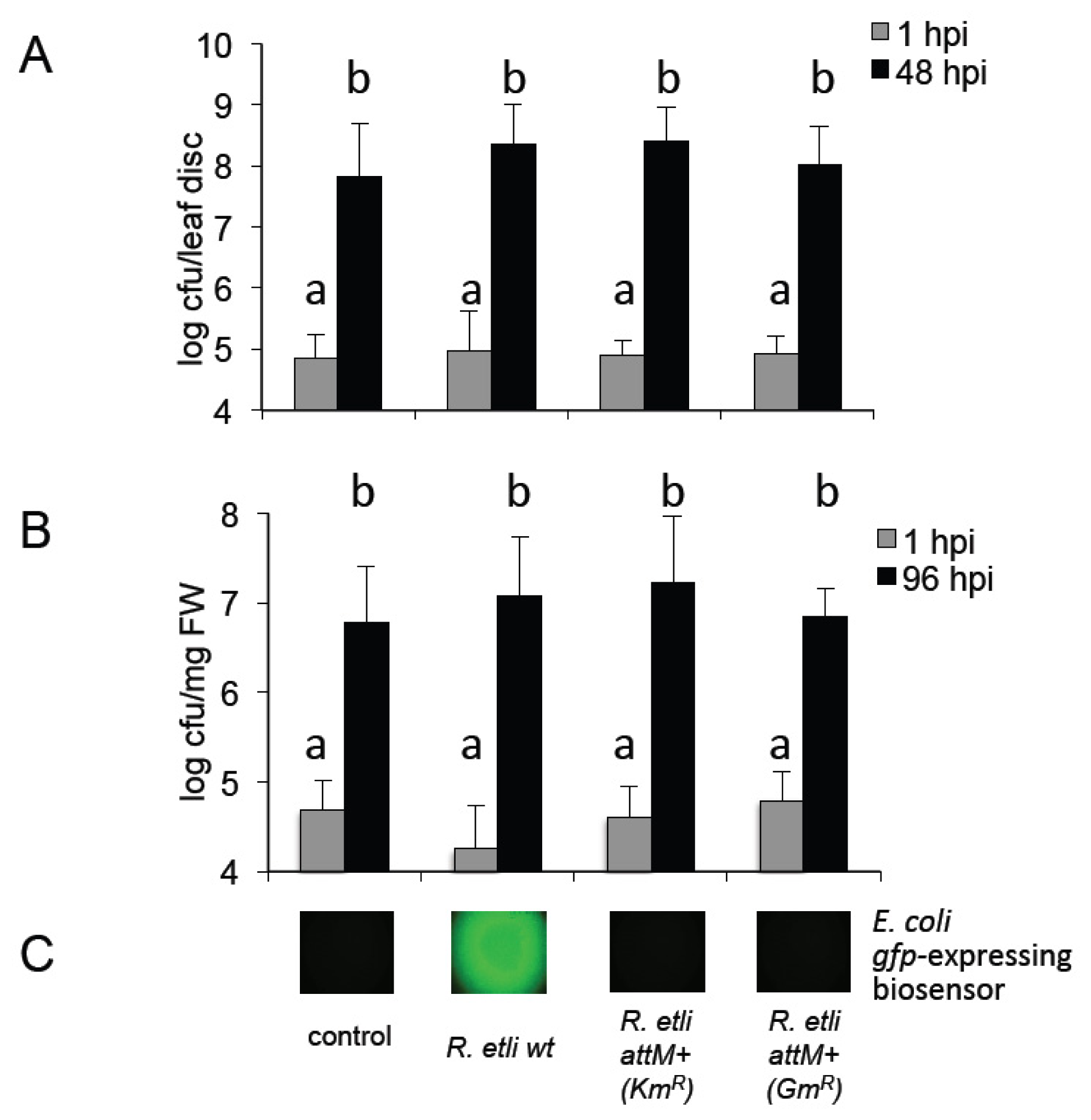

Supplement: Figure S3 — R. etli strains have no impact on Arabidopsis resistance towards P. syringae bacteria. Proliferation of the plant pathogenic P. syringae DC3000 (Pst) on plants pretreated with MgCl2 (control) or different R. etli strains, producing different quantities of oxo-C8-HSL. (A) Pst bacteria were infiltrated into Arabidopsis leaves. OD600 nm = 0.01; (B) Arabidopsis plants were spray-inoculated with Pst bacterial solution, OD600 nm = 0.1; (C) AHL production in R. etli strains used in the above experiments detected by the bacterial biosensor strain of E. coli. Student’s t-test revealed no differences between the treatments at p ≥ 0.05, as indicated by the letters in (A) and (B). [file ijms-14-17122s3.tif]

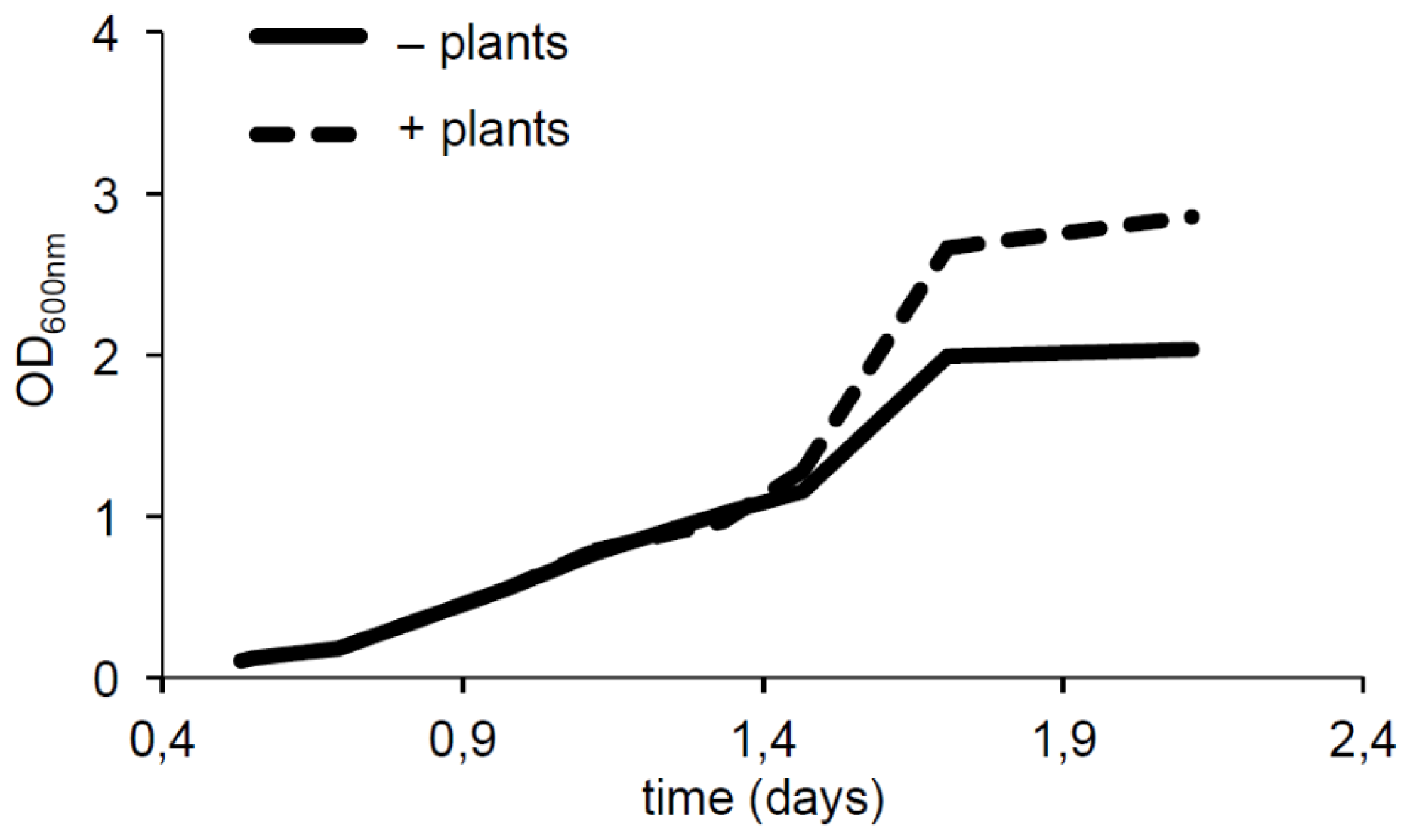

Supplement: Figure S4 — Growth of S. meliloti in the presence of Arabidopsis plants. Proliferation of S. melioti measured over the period of 2 days in medium with or without Arabidopsis seedlings. [file ijms-14-17122s4.tif]
